# Coumarin-Induced Hepatotoxicity: A Narrative Review

**DOI:** 10.3390/molecules27249063

**Published:** 2022-12-19

**Authors:** Michele Pitaro, Nicoletta Croce, Valentina Gallo, Alyexandra Arienzo, Giulia Salvatore, Giovanni Antonini

**Affiliations:** 1 INBB—Biostructures and Biosystems National Institute, Viale delle Medaglie d’Oro 305, 00136 Rome, RM, Italy; 2 Department of Science, Roma Tre University, Viale Guglielmo Marconi 446, 00146 Rome, RM, Italy

**Keywords:** coumarin, 1,2-benzopyrone, *Melilotus officinalis*, narrative review, primary lymphoedema, secondary lymphoedema, hepatotoxicity

## Abstract

Coumarin is an effective treatment for primary lymphoedema, as well as lymphoedema related to breast cancer radiotherapy or surgery. However, its clinical use is limited in several countries due to the possible occurrence of hepatotoxicity, mainly in the form of mild to moderate transaminase elevation. It is worth noting that only a few cases of severe hepatotoxicity have been described in the literature, with no reported cases of liver failure. Data available on coumarin absorption, distribution, metabolism, and excretion have been reviewed, focusing on hepatotoxicity studies carried out in vitro and in vivo. Finally, safety and tolerability data from clinical trials have been thoroughly discussed. Based on these data, coumarin-induced hepatotoxicity is restricted to a small subset of patients, probably due to the activation in these individuals of alternative metabolic pathways involving specific CYP450s isoforms. The aim of this work is to stimulate research to clearly identify patients at risk of developing hepatotoxicity following coumarin treatment. Early identification of this subset of patients could open the possibility of more safely exploiting the therapeutical properties of coumarin, allowing patients suffering from lymphoedema to benefit from the anti-oedematous activity of the treatment.

## 1. Historical Background

By the end of the nineteenth century, breeders in North America began to sow *Melilotus officinalis* and *Melilotus alba* (Sweet clover) imported from Europe to feed their livestock [1]. Soon after, cattle began to develop a new and lethal disease characterized by profuse bleeding [2]. Francis Schofield, an English veterinarian who emigrated to Canada, guessed that the disease was linked to the consumption of spoiled hay, since fresh hay caused no disease. He demonstrated that the elimination of spoiled hay from the diet, as well as blood transfusion from healthy animals, significantly improved the health condition of the affected animals [3].

The etiopathogenesis of the new disease remained a puzzling question, until Karl Paul Link and his colleagues at the University of Wisconsin discovered that spoiled hay was contaminated by several species of Aspergillus [2]. These fungi oxidize the coumarin naturally present in *Melilotus* into 4-hydroxycoumarin, which in turn reacts with formaldehyde and another molecule of coumarin, leading to the production of dicumarol [4]. Link also demonstrated that bleeding induced by spoiled *Melilotus*, as well as dicumarol, was antagonized by the administration of vitamin K, which promotes blood clotting [5]. Patent rights on dicumarol were transferred to the Wisconsin Alumni Research Foundation, which in turn licensed the patent to Lilly, Squibb, and Abbott for the treatment of thrombosis and myocardial infarction [6].

In 1945, Link was hospitalized in a sanatorium, where he spent some time trying to identify an efficient rat poison [7]. He thought that an ideal substance should induce a slow death, otherwise rodents would associate product consumption with its lethal effects. Bearing in mind the haemorrhagic disease in livestock, Link began to test dicumarol in rodents. However, dicumarol turned out to be less toxic in rodents compared with cattle. For this reason, he examined a series of compounds synthesized a few years before by a group of Japanese researchers, focusing his attention on a specific compound named 3-phenyl-acetyl ethyl-4-hydroxycoumarin [8]. The product turned out to be an effective rat poison and Link again transferred the patent rights to the Wisconsin Alumni Research Foundation, calling the new product “warfarin” from the foundation’s acronym [6].

Following its launch on the market as a rat poison, Link convinced some clinicians to test warfarin also as a therapeutic agent in humans [9]. Clinical studies showed that warfarin was superior to dicumarol as an anticoagulant, and when US President Eisenhower suffered a myocardial infarction in 1954, he was successfully treated with warfarin [10]. Still now, the product is marketed under the trade name “Coumadin”, generating confusion between dicumarol derivatives and coumarin. However, while the formers are potent anticoagulants, the latter is completely devoid of anticoagulant activity.

## 2. Introduction

Natural coumarins are generally unsaturated lactones, with an oxygenated substituent in position 7, biosynthesized from phenylalanine via the shikimic acid [11,12]. They are classified into six main types based on their structure: simple coumarins, furocoumarins, dihydro-furocoumarins, pyranocoumarins, phenyl-coumarins, and bis-coumarins [13,14].

**Figure 1:**
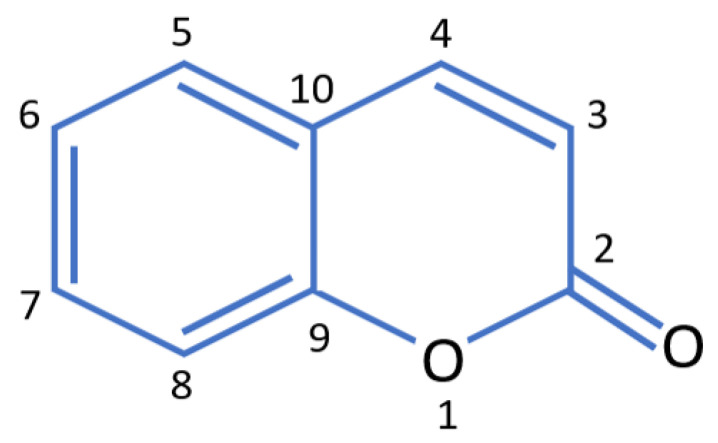
Structure of coumarin (1,2-benzopyrone).

The name “coumarin” refers exclusively to the simplest representative of these compounds, the 1,2-benzopyrone (or 5,6-benzo-[α]-pyrone). From a chemical point of view, coumarins are aromatic heterocyclic compounds belonging to the family of benzopyrones; their structure consists of a benzene ring fused to an α-pyrone ring [13,15] (Figure 1).

Coumarins are secondary metabolites of numerous species of higher plants, including *Melilotus officinalis* (sweet clover) and other different species (spp.), *Angelica keiskei* (ashitaba), *Angelica pubescens* (pubescent angelica), *Artemisia scoparia* (yin-chen wormwood), *Citrus* spp. (orange), *Glycyrrhiza uralensis* (licorice), *Justicia pectoralis* (chambá), *Mikania glomerata* (guaco), *Pelargonium sidoides* (African geranium), *Leonurus heterophyllus* (Chinese motherwort), *Cinnamomum aromaticum* (cassia) and *Cinnamomum zeylanicum* (true cinnamon) [16]. Coumarins are also produced by some species of bacteria, fungi, and sponges [17,18], and can be obtained by synthetic processes [19].

**Figure 2:**
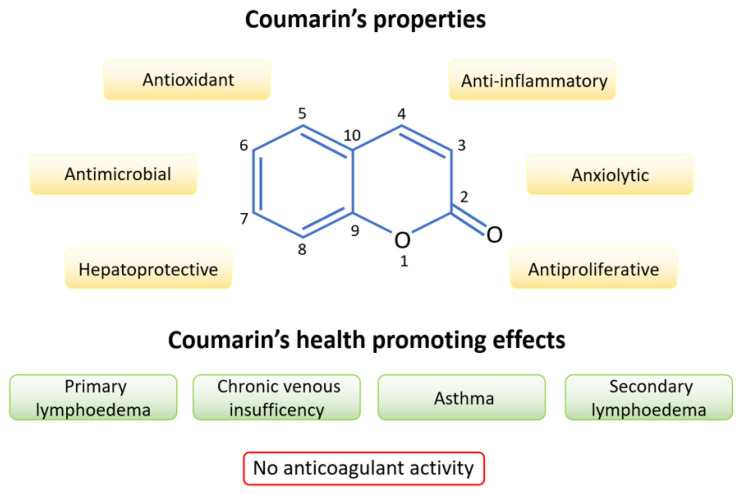
Coumarin’s biological properties and therapeutical applications.

Coumarin (1,2-benzopyrone) is a phytochemical known to exert diverse biological and pharmacological activities [20] that render this molecule very promising in a wide spectrum of applications, including medical and agrochemical fields as well as the cosmetic industry [21,22,23,24]. Coumarin is characterized by anti-inflammatory [25], antioxidant [26], hepatoprotective [27], anxiolytic [28], antimicrobial, and antiproliferative properties (Figure 2) [29]. Contrary to some natural coumarin family members, such as dicumarol, and synthetic coumarins (i.e., warfarin) that are vitamin K antagonists [30,31], coumarin is completely devoid of anticoagulant effects [11,31].

The first studies addressing the use of coumarin in the treatment of lymphoedema, a high-protein oedema caused by a failure of the lymphatic system, date back to the 1970s [32,33]. The effectiveness of coumarin in the treatment of both primary and secondary lymphoedema was also confirmed by more recent studies that showed its role in significantly increasing the reabsorption rate [34]. Most of these studies suggested that this activity is mainly due to the coumarin metabolite 7-hydroxycoumarin [35]. Furthermore, data suggest coumarin-mediated macrophages activation and recruitment at the level of target tissues as a putative mechanism of action. Two different mechanisms have been hypothesized: (1) macrophages activation leads to phagocytosis of coumarin and coumarin-bound plasma proteins at the level of microvascular vessels, resulting in a decrease of colloid pressure in the intercellular spaces, and consequently in a decrease of lymphoedema volume; (2) coumarin-activated macrophages stimulate the release of lysosomal enzymes, increasing proteolysis [36]. These data have been the basis for several clinical trials carried out in the past years on primary lymphoedema, as well as on lymphoedema due to radiotherapy and surgery in cancer patients.

In addition to lymphoedema, the health-promoting actions of coumarin have been demonstrated in other diseases including chronic venous insufficiency, asthma, and breast cancer [37,38]. These results, together with coumarin’s bioavailability and low cost, make this compound very attractive as a therapeutic agent; its great versatility could be exploited in diverse fields including pharmacology, but also medicinal chemistry [39], and food science [40].

Despite its promising features, early after its first isolation (by Vogel in 1820, from the seeds of Dipteryx odorata) [41], coumarin was noted to cause hepatic damage in animal models [42] and, later, also to induce long-term tumour formation in rodents [43]. Coumarin-induced carcinogenesis was demonstrated not to be related to genotoxicity [44,45], but to its sub-acute and chronic toxic effects, especially hepatotoxicity, confirming previous studies [46]. Considering this, coumarin hepatotoxic effects were the most extensively studied, not only in rodents but also in other mammalian species.

In humans, the onset of coumarin-induced hepatotoxicity is extremely rare. However, several clinical studies demonstrated a possible correlation between coumarin treatment and hepatotoxicity, usually in the form of a significant increase in transaminase level, in a very small subgroup (single-digit percentage) of patients [47]. Indeed, elevation of transaminases levels, especially levels of ALT > 3 times the ULN, is a strong indicator of drug-induced hepatotoxicity [48]. Even if the mechanisms are still not completely clarified, it is interesting to note that studies demonstrated an association with high-energy reactions involving cytochrome P-450 enzymes, causing decay of adenosine triphosphate levels, loss of ionic gradients, cell swelling and damage [49].

Studies demonstrated that genetic variability, especially in the expression of CYP2A6, an enzyme involved in the metabolism of coumarin [50], and environmental factors can significantly induce inter-individual variations in the metabolism of coumarin and modulate the individual response to the drug [41]. Due to this, further studies to determine coumarin safety are needed. Importantly, research should mainly focus on the individuation of the susceptibility factors that make some individuals vulnerable to coumarin toxicity. The identification of idiosyncrasies can be crucial not only to protect vulnerable patients, but also to fully exploit coumarin as a pharmaceutical for patients that are not at risk of developing hepatotoxicity and that could greatly benefit from this treatment. Among the diverse possible strategies to reach this aim, pharmacogenetics, together with new integrated methodologies, have recently been considered very promising approaches, as reported by Hu et al. [34].

In the last few years, literature specifically focused on coumarin addressed hepatotoxicity only marginally, and in most cases referred to outdated studies. Several papers have been recently published on the use of coumarin in the treatment of diverse pathologies, which include most of the available data on hepatotoxicity. However, none of the recent clinical studies addressed hepatotoxicity specifically [13,51,52,53,54,55,56,57,58]. An interesting recent review gives a thorough overview of the molecular interactions between CYPs and coumarin and their significance in pharmacology and toxicology [59]. However, to our knowledge, no review also focuses on the clinical evidence supporting or disproving the hepatotoxic effects of coumarin in humans.

The purpose of this narrative review is to summarize what is known to date about coumarin hepatotoxicity, with a focus on the possible strategies to identify subjects at risk of developing this complication. This work aims to stimulate research in this field; indeed, the individuation of subject at risk of hepatotoxicity could open the possibility to more safely exploit the therapeutical potential of coumarin.

## 3. Results

### 3.1. Absorption and Distribution

Coumarin is completely absorbed following oral administration. However, only approximately 2–6% of coumarin reaches systemic circulation in its native form, while plasma levels of its main metabolite, 7-hydroxycoumarin-glucuronide (7-HCG), rise significantly and proportionally after administration [60]. Coumarin is also absorbed through the skin; in a 70% aqueous ethanol solution the overall amount of absorbed product reaches 60% of the applied quantity in humans after 72 h, a percentage that increases if the skin is immediately occluded after exposure [61]. Coumarin’s half-life in the blood ranges between 1 and 1.5 h. The biological half-life of both coumarin and 7-HCG does not vary between oral or intravenous administration [62]. Coumarin is considered a pro-drug since the active form is 7-hydroxycoumarin.

Coumarin distribution in the body follows two-compartment kinetics. The high distribution volume (1.7 times the body weight) is explained by the fact that coumarin and its metabolites are found not only in organs with high blood flow, but also in extracellular and intracellular compartments [63]. Early studies hypothesized that cells could store coumarin, but pharmacokinetic studies showed that this is not the case [50].

### 3.2. Metabolism and Excretion

**Figure 3:**
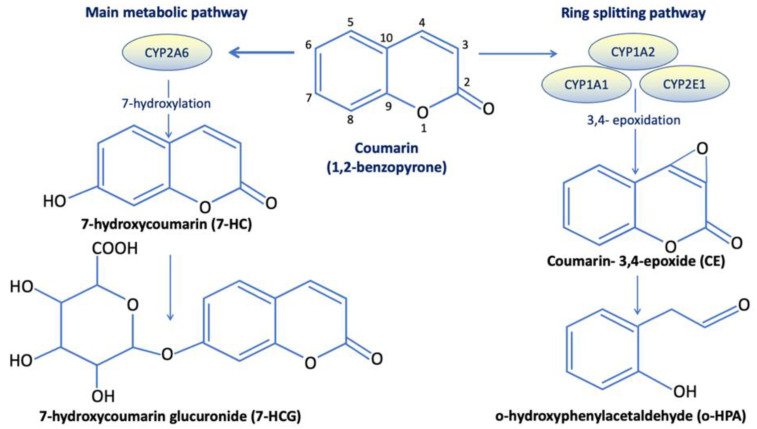
Coumarin metabolic pathways and its main metabolites in humans. Coumarin can be metabolized either through the main metabolic pathway mediated by cytochrome P450-linked mono-oxigenase enzyme system (CYP2A6) (**left**) or through the uncommon ring-splitting pathway, mediated by other cytochrome P450 isoforms (namely CYP1A1, CYP1A2, and CYP2E1), which leads to the production of the hepatotoxic metabolite o-HPA (**right**).

Following intestinal absorption, coumarin reaches the liver through the portal circulation. About 97% of the coumarin absorbed is metabolized by the cytochrome P450-linked mono-oxygenase enzyme system (CYP2A6) in liver microsomes, which performs hydroxylation [51,64,65,66]. Although hydroxylation could potentially occur on each carbon (i.e., 3, 4, 5, 6, 7, and 8), 7-hydroxycoumarin is the main metabolite. The 7-hydroxycoumarin is conjugated in the gut and other tissues to glucuronic acid (and to a lesser extent to sulphate), producing 7-hydroxycoumarin-glucuronide. In a separate and rarely occurring metabolic pathway, coumarin is metabolized by other cytochrome P450 isoforms (namely CYP1A1, CYP1A2, and CYP2E1) via a ring-splitting pathway into a highly unstable compound called 3,4-epoxycoumarin (CE). CE can either rearrange spontaneously to o-hydroxyphenylacetaldehyde (o-HPA) or be conjugated with glutathione (GSH) [20,41]. o-HPA is a hepatotoxic aldehyde and can be further detoxified by oxidation to o-hydroxyphenylacetic acid (o-HPAA) [67]. Lewis et al. [68] reviewed studies on the metabolism catalysed by human P450 enzymes, and reported that P450 isoforms CYP1A1, CYP1A2, CYP2B6, CYP2E1, and CYP3A4 could all catalyse the metabolism of coumarin along the 3,4-coumarin epoxide pathway, whereas CYP2A6 catalyses exclusively the formation of 7-hydroxycoumarin (Figure 3).

The main metabolite of coumarin, 7-hydroxycoumarin-glucuronide, is actively secreted in the renal tubules and accounts for approximately 60% of the ingested dose of coumarin. However, coumarin is also present in its free and sulphated forms, as o-HPAA is. The latter is a minor metabolite in humans but is found in greater amounts in mouse (41% of the administered dose) and rat (12% of the administered dose) urines [69].

### 3.3. In Vitro and In Vivo Studies

The first reports on coumarin toxicity in rats and dogs date back to the 1950s, and prompted several studies on coumarin metabolism in the liver. While coumarin-induced hepatotoxicity has been observed in rats [43], coumarin is not toxic in other rodents, such as mice, hamsters, and gerbils [70,71]. Differences observed among species, regarding coumarin hepatotoxicity, have been demonstrated to be metabolism-mediated. In most species, coumarin is hydroxylated to 7-hydroxycoumarin (7-HC), a nontoxic metabolite [72,73,74]. In rats, however, the formation of 7-HC is extremely low [75,76], and this is thought to make rats more susceptible to hepatotoxicity [43], since the 3–4 epoxidation pathway is prevalent. Importantly, coumarin metabolism differs significantly in rats and humans. For this reason, rat’s suitability for studying risk associated with coumarin intake in humans has been questioned [77]. Indeed, several clinical trials carried out in humans indicate that coumarin hepatotoxicity develops only in a small subset of treated patients [78,79,80,81,82].

In humans, the formation of 7-HC by CYP2A6 is the predominant metabolic pathway, while only a minor amount of coumarin follows the alternative pathway that leads to the activation of the epoxide intermediate CE. CE can rearrange to form o-HPA. It is hypothesized that this is the prevailing metabolic pathway in patients that develop hepatotoxicity. In this context, it has been hypothesized that CYP2A6 polymorphisms decreasing its enzymatic activity [83,84,85] could shift coumarin metabolism towards the production of CE and o-HPA and could represent a possible risk factor for coumarin-induced hepatotoxicity [84,86]. Farinola and Piller suggested that a reduction in coumarin 7-hydroxylation could indeed lead to its toxicity, and that subjects with low levels of CYP2A6 activity are more likely to metabolize coumarin via the cytotoxic pathway [87]. To date, 34 CYP2A6 polymorphisms have been identified. Most of the mutations significantly decrease enzymatic activity, however the variants CYP2A6*28 and CYP2A6*31 show the same activity as the “wild type” enzyme. On the other hand, variants CYP2A6*14 and CYP2A6*15 are characterized by a higher enzymatic activity. These data suggest that not all CYP2A6 polymorphisms lead to a decrease in enzymatic activity [88], and therefore to a putative higher susceptibility to coumarin hepatotoxicity.

A study carried out by Van Iersel et al. [89] using a panel of human liver microsomal samples of known P450 isoenzyme profile demonstrated a 30- to 2250-fold variation in coumarin metabolism to total polar products (i.e., all metabolites except products covalently bound to microsomal proteins) and 7-HC. The authors observed that a marked interindividual difference exists in coumarin metabolism by human liver microsomes, hypothesizing that subjects with low levels of CYP2A6 activity may metabolize coumarin by the 3-hydroxylation and other pathways. To test this hypothesis, a clinical trial on 231 patients treated with coumarin or placebo was carried out in Germany [90]. In this study, patients were genotyped for the two allelic variants encoding the defective proteins CYP2A6*2 and CYP2A6*3. The authors determined that susceptibility to coumarin-associated liver dysfunction is not genetically determined by polymorphisms in CYP2A6, concluding that the studied polymorphisms are not the primary reason for coumarin hepatotoxicity. In addition, Rietjens et al. noted that both the peak concentration and the area under the curve of o-HPA in the human liver after 24 h is always significantly lower than that observed in the rats [91]. This observation is valid in subjects with normal (“wild type”), as well as decreased CYP2A6 activity. In other words, even in the case of CYP2A6 deficiency (a phenomenon that occurs in less than 1% of Caucasian populations and about 20% of Asian populations) [92], the production of o-HPA in the human liver is lower than in the rat liver.

These data suggest that a reduction in the CYP2A6 metabolic pathway should not be responsible alone for the observed cases of hepatotoxicity. However, this does not exclude that different factors could shift coumarin metabolism, leading to CE-derived hepatotoxic compound accumulation. Indeed, metabolic activation of coumarin to CE through the heterocyclic ring-splitting pathway is an important prerequisite of toxicity. Dihydrocoumarin, which lacks the 3,4-double bond, is not hepatotoxic, as well as the analogues of coumarin with substitutions on the 3,4 double-bond [93,94].

The hypothesis that epoxidation alone is responsible for hepatotoxicity is nevertheless partially disproved by in vitro analysis carried out on the kinetics of coumarin epoxidation in rat and mouse liver microsomes. These analyses indicated that hepatic clearance of coumarin through the epoxide intermediate is about four times greater in mice than in rats [95]. However, mice show little or no hepatotoxicity after coumarin treatment. The lack of a direct correlation between coumarin epoxidation and species sensitivity to hepatotoxicity suggests that factors other than metabolic activation to CE are important determinants of hepatotoxic outcome. Studies suggested that the CE detoxification process could play a role in this outcome. Indeed, CE can be conjugated with GSH or rearrange to o-HPA, a toxic compound that can be detoxified by further oxidation to o-HPAA or reduction to o-HPE [76,96,97,98]. Vassallo et al. showed that the oxidation of o-HPA to o-HPAA seems to be crucial in determining hepatotoxicity of coumarin. Major differences in this reaction among species were observed. The clearance of o-HPA through this pathway proceeds more than 20 times faster in mice than in rats. This is consistent with the observation that in mice, all the o-HPA formed was oxidized to o-HPAA, whereas in rats, o-HPA remained as a major component detected in the microsomal reaction mixture. The slower hepatic clearance of the toxic aldehyde appears to be responsible for coumarin-induced hepatotoxicity in rats [99]. However, authors did not investigate the potential shift of the metabolic pathways in humans with polymorphisms in which 7- hydroxycoumarin formation is blocked, and whether this might be linked to hepatotoxicity in humans.

Regarding human hepatic metabolism of coumarin, oxidation of o-HPA to o-HPAA was higher compared with GSH conjugation of CE, and clearance of o-HPA through the oxidation pathway was considerably faster (more than 50 times) compared with rats [99]. This means that, in the human liver, the conversion to CE is likely to be very low, and the oxidation of o-HPA to o-HPAA occurs efficiently, leading to the low hepatotoxicity of coumarin in humans.

### 3.4. Clinical Trials

Coumarin has been used since the 1970s for the treatment of various pathologies, including lymphoedema, varicose veins, lung and kidney carcinoma, melanoma, infections, and chronic fatigue syndrome [100,101,102,103,104,105,106,107]. Thus, thousands of individuals have been exposed to therapeutic doses of coumarin for periods ranging from 2 weeks to over 2 years. Recommended doses range from 8 mg for the treatment of venous constriction to 7000 mg/day in antineoplastic therapies [37]. These are doses up to 2000 times higher than the estimated maximum daily intake of coumarin, calculated considering oral and dermal exposure [108].

Several clinical studies have investigated the occurrence of hepatotoxicity in patients treated with therapeutic doses of coumarin [78,79,80,81,82,109]. Overall adverse reactions linked to hepatotoxicity, such as elevated liver enzymes in serum and clinical hepatitis, were addressed only in a small proportion of patients. Those patients usually displayed liver alterations that reverted to normal after cessation of treatment, while liver failure occurred only in extremely rare cases [43]. However, for this reason, coumarin has been withdrawn from the market in France and other countries [110].

Marshall et al. have published three papers on the use of coumarin in combination with cimetidine for the treatment of several metastatic cancers, such as non-small cell lung cancer, renal cell carcinoma, and melanoma [105,106,111]. These studies were subsequently grouped into a single publication on the effects of coumarin for the treatment of advanced malignancies. Overall, 91 patients (24 with non-small cell lung cancer, 45 with renal carcinoma, and 22 with melanoma) were treated with 100 mg of coumarin together with 300 mg of cimetidine daily. No cases of hepatotoxicity were reported by the authors [37].

Mohler et al. carried out a clinical trial in the United States on 48 patients with prostate cancer treated with 3 g per day of coumarin [112]. The authors reported limited treatment hepatotoxicity, with three patients developing asymptomatic transaminase increase; only mild adverse reactions (such as nausea with vomiting) were assessed in an additional four patients, without any sign of hepatotoxicity.

Casley-Smith et al. carried out a randomized, crossover, double-blind clinical trial in Australia on 31 patients with upper-limb lymphoedema secondary to breast cancer and 21 patients with primary lower-limb lymphoedema, treated for the first six months with placebo and for the following six months with 400 mg per day of coumarin [113]. Apart from a few cases of transient gastrointestinal discomfort (nausea and diarrhoea), the authors did not report any case of transaminase elevation.

An additional clinical trial on 104 patients with chronic lymphatic filariasis enrolled in the Shandong province of China was carried out by Casley-Smith et al. [114]. Of these, 45 patients were randomized to receive 400 mg per day of coumarin and 38 to receive placebos for 367 days. In this study, about 60% of coumarin-treated patients experienced mild symptomatology as dizziness or drowsiness that, however, disappeared after the first month of treatment. No correlation between these symptoms and hepatotoxicity was revealed. Blood and urine tests were found to be normal and, in particular, there were no elevations in transaminase levels or, more generally, alterations in liver function parameters.

In a total of five clinical trials including 1106 lymphoedema patients treated with a daily dose of 400 mg of coumarin for a mean duration of 14.6 months, Casley-Smith et al. [79] reported two cases of hepatotoxicity (incidence 0.18%). In one case, symptoms regressed immediately after stopping treatment, while the symptomatology of the second patient was successively related to other causes.

In another clinical trial performed on 2173 patients with cancer or chronic infections treated with a daily dose of coumarin ranging from 25 to 2000 mg, with a majority receiving 100 mg per day for one month and then 50 mg per day for two years, eight patients (0.37%) developed elevated liver enzymes (serum transaminases) after total doses of between 1 and 15 g of coumarin [103].

Morrison and Welsby [80] were the first to report a severe hepatic reaction to coumarin (characterized by high transaminases levels, malaise and icterus), in a lymphoedema patient treated with 400 mg of coumarin daily for five months. All abnormalities, however, resolved five weeks after the treatment when coumarin was discontinued. Later, Koch et al. [82] reported two cases of acute hepatitis in patients treated with 90 mg/d of coumarin for 5 months. Authors observed a marked increase in serum aminotransferases (ALT: 30 and 100 times higher than the upper limit of the physiological range) in conjunction with clinical features including jaundice, pruritus, and diarrhoea. Coumarin withdrawal was rapidly followed by a favourable outcome in both cases.

Burgos et al. carried out a double-blind clinical trial in Spain on 77 women aged 35 to 65 with upper limb lymphoedema secondary to radiotherapy or surgery for breast cancer [115]. Patients were randomized to receive coumarin at a dose of 90 mg per day (38 women) or 135 mg per day (39 women) for 12 months. A patient treated with 90 mg per day of coumarin (2.63%) with normal SGPT levels at baseline (15 U/L) showed a significant increase after 6 months (107 U/L). Similarly, a patient treated with 135 mg per day of coumarin (2.56%) with normal SGPT levels at baseline (47 U/L) showed a significant increase after 6 months (82 U/L). The authors reported that, in both cases, SGPT levels decreased after 12 months of treatment (respectively to 43 and 56 U/L). In all these cases, symptoms were reversible and ceased after termination of coumarin treatment.

In other trials performed on 50 [104] and 17 [116] cancer patients treated with 100 mg per day of coumarin in association with 1 g per day of cimetidine, no evidence of liver toxicity was observed.

Jamal et al. carried out a double-blind, placebo-controlled, randomized clinical trial in India on 169 patients with chronic lymphoedema secondary to filariasis [100]. Of these, 42 patients were treated with 400 mg of coumarin and 6 mg/kg of diethylcarbamazine per day; 39 patients were treated with placebo and 6 mg/kg of diethylcarbamazine per day; 47 were treated with 400 mg per day of coumarin and placebo; and finally, 41 patients were treated with placebo of both products. An interim analysis conducted when the average duration of treatment was 9.3 months and 20 patients had completed the two years of treatment reported no cases of hepatotoxicity. Only mild adverse reactions were recorded, mainly abdominal pain, diarrhoea, constipation, and dizziness, with no significant difference between the four study groups.

Thornes et al. recruited 29 patients with melanoma undergoing surgical resection. Among these, 13 patients were treated with 50 mg per day of coumarin until disease progression. Treatment tolerability was good in all patients and no adverse reactions were recorded, including a patient that assumed coumarin during pregnancy [102].

Kokron et al. carried out a clinical trial in Austria on 38 patients with metastatic renal cell carcinoma and one patient with a second primary renal cell carcinoma treated with 100 mg of coumarin and 400 mg of cimetidine per day until disease progression. One patient discontinued treatment due to the onset of nausea correlated to coumarin intake by the authors. No hepatotoxicity was registered [117].

More recently, Grötz et al. investigated the efficacy of coumarin in combination with troxerutin for the protection of salivary glands and mucosa during radiotherapy in 48 patients with head and neck cancer recruited in Germany in a randomized, double-blind, placebo-controlled clinical trial. The treatment schedule included the administration of 90 mg of coumarin and 540 mg of troxerutin per day for five weeks. No adverse events were correlated to the combination of the two products [118].

Vanscheidt et al. recruited 231 patients with lower-limb oedema secondary to chronic venous insufficiency in a randomized, double-blind, placebo-controlled clinical trial in Germany. Of these, 114 were treated with 90 mg of coumarin and 540 mg of troxerutin daily for 16 weeks. Liver function parameters (ALT, AST, alkaline phosphatase, and gamma-GT) were monitored at baseline and after 4, 6, 8, 12, and 16 weeks of treatment. No hepatotoxicity was reported, while minor reactions were found in 25 cases (21.9%) in the coumarin and troxerutin group versus 14 cases (12.0%) in the placebo group. The relative risk of developing elevations greater than 1.25 times the physiological range of major liver function parameters (GGT, ALT, and AST) was 4.9% in the active treatment group compared with 2.1% in the placebo group. The values returned to normal, sometimes during the treatment, in other cases after treatment discontinuation. Overall, authors reported a high drug tolerability both in the active treatment group and in the placebo group [119]. These results were confirmed by a separate publication focused on safety assessment which concluded that coumarin treatment is safe and well-tolerated [120].

Lessiani et al. carried out an open-label clinical trial in Italy on 60 patients suffering from lymphoedema of the lower limbs secondary to surgery. Of these, 36 were randomized to receive one tablet per day of a product containing 50 mg of *Melilotus officinalis* extract (corresponding to 10 mg of coumarin), 50 mg of *Vitis vinifera*, and 200 mg of troxerutin for 30 days in addition to prophylaxis with heparin and the use of elastic stockings. No hepatotoxic effects were reported, and the number of other adverse reactions in the active treatment group did not differ significantly from those recorded in the control group [121].

Although the above-discussed studies agree in reporting that coumarin causes hepatotoxic effects in fewer than 1% of patients, and an association between coumarin and hepatotoxicity cases was not clearly documented, the only discording and unexpected result was obtained by Loprinzi et al. In their study, the authors recruited 140 patients in the United States with lymphoedema secondary to radiotherapy or surgery for breast cancer [122]. Patients received either 400 mg per day of coumarin or placebo for six months, followed by six months with the alternative treatment in a randomized, cross-over clinical trial. There were no significant differences in the incidence of nausea, vomiting, or diarrhoea during treatment with coumarin or placebo, but the incidence of hepatotoxicity was higher during treatment with coumarin. Nine women (corresponding to 6%) experienced significant transaminase increase (*p* = 0.006) which promptly regressed upon treatment discontinuation. One woman developed jaundice with bilirubin levels reaching 19.3 mg/dL.

Table 1 summarizes the main safety and tolerability data of coumarin in the clinical studies described above.

Based on these studies, coumarin-induced hepatotoxicity can be considered rare; most of the cases have been reported as idiosyncratic or due to an unpredictable adverse drug reaction affecting a small subgroup of the population. Except for Loprinzi et al. [122], most of the above-mentioned clinical studies report that there is no clear relationship between coumarin and hepatotoxicity and that any changes in liver function parameters are transient. Moreover, a relationship between coumarin dose and hepatotoxicity has not been clearly demonstrated. Indeed, the time to onset of hepatotoxicity varied from 1 to 6 months, with the lowest dose observed to cause adverse effects of 87 mg/kg bw for male patients and of 30 mg/kg bw for female patients, both with oral dosing [103]. In patients treated with less than 25 mg per day, no liver toxicity has been reported [47]. Overall, coumarin can be considered safe and well-tolerated in the majority of the treated patients.

## 4. Materials and Methods

The research was carried out on two different databases, i.e., Medline (Pubmed) and EMBASE (Elsevier), using the following search strings:

- “coumarin” OR “1,2-benzopyrone” OR “5,6-benzo-[+]-pyrone” AND “hepatotoxicity”
- “coumarin” OR “1,2-benzopyrone” OR “5,6-benzo-[+]-pyrone” AND “ADME”
- “coumarin” OR “1,2-benzopyrone” OR “5,6-benzo-[+]-pyrone” AND “metabolism” AND “human” (last 10 years)
- “coumarin” OR “1,2-benzopyrone” OR “5,6-benzo-[+]-pyrone” AND “human cell line”
- “coumarin” OR “1,2-benzopyrone” OR “5,6-benzo-[+]-pyrone” AND “clinical trial”
- “coumarin” OR “1,2-benzopyrone” OR “5,6-benzo-[+]-pyrone” AND “case report”
- “coumarin” OR “1,2-benzopyrone” OR “5,6-benzo-[+]-pyrone” AND “observational trial”
- “coumarin” OR “1,2-benzopyrone” OR “5,6-benzo-[+]-pyrone” AND “observational study”

Paper selection was carried out by three authors. For each search engine, two authors carried out the preliminary selection, involving the third author in case of disagreement. Additional papers were selected from the bibliography of previously selected papers.

Finally, the paper was sent to an independent reviewer (Prof. Neil Piller, Director of the Lymphoedema Clinical Research Unit at the Department of Surgery, College of Medicine and Public Health of the Flinders University and Medical Centre, South Australia) who kindly revised the manuscript, provided his valuable advice, and suggested some articles not included in our selection.

## 5. Discussion

On the 4th of October, Swedish people celebrate the “Kanelbullens Dag” (The Cinnamon Bun Day). Kanelbullens are characteristic cinnamon-flavored pastries very popular in Sweden. Most of the cinnamon on the market comes from China (Cinnamomum cassia) and contains significant amounts of coumarin (2.23 mg/g dry weight). True cinnamon (Cinnamomum zeylanicum) from Sri Lanka would be a better alternative, since its content of coumarin is negligible (<0.01 mg/g dry weight). However, true cinnamon is quite expensive and, for this reason, it is not commonly used in bakery [124].

In 2004, the Scientific Panel on Food Additives, Flavourings, Processing Aids and Materials in Contact with Food of the European Food Safety Authority (EFSA) adopted an opinion in which it was concluded that coumarin was not genotoxic in experimental animals, allowing the derivation of a Tolerable Daily Intake (TDI) [109]. Taking into consideration that the most sensitive animal species were rats and dogs, based on a two-year dog study, the overall No Observed Adverse Effect Level (NOAEL) for liver toxicity was found to be 10 mg/kg bw per day. Applying a total safety factor of 100 to this NOAEL (a factor of 10 for potential interspecies variation, together with a factor of 10 for potential interindividual differences in humans), it was concluded that a TDI of 0–0.1 mg coumarin/kg bw could be established [109,125]. This conclusion was further supported by the German Federal Institute for Risk Assessment (BfR) [126,127]. Based on the data of Bergmann et al. [108], the BfR considered 25 mg to be the lowest dose capable of inducing an hepatotoxic response and applied an extrapolation factor of 5 (assuming a typical slope of the dose-response curve), resulting in a level of 5 mg of coumarin per day. This dose is expected to cause no adverse effects, even in sensitive people, confirming the TDI of 0.1 mg/kg bw. Considering the toxicity data of coumarin, including the timing to onset of liver effects, recovery of these effects after cessation of treatment, and the elimination half-life, international committees have concluded that exposure to coumarin resulting in an intake three times higher than the TDI for one to two weeks is not of safety concern.

On the basis of the above-mentioned opinions, Kanelbullens should have been banned from the European Union. However, the adoptions of these opinions in the national legislations led to the protest of bakers in Sweden and Denmark in the so-called “cinnamon-gate” [128]. As a matter of fact, Kanelbullens do not seem to represent a serious threat to the health of consumers, apart from very rare cases of anaphylaxis to the wheat proteins [129] and their high content in butter (not the ideal food to maintain an appropriate weight).

According to the EU Directive 2002/46/EC, “food supplements’ means foodstuffs the purpose of which is to supplement the normal diet and which are concentrated sources of nutrients or other substances with a nutritional or physiological effect, alone or in combination”. As clearly stated in the Directive, “nutrients means the following substances: vitamins and minerals.” [130]. Based on this definition, coumarin would not fall in the category of food supplements.

In 1995, De Felice coined the term nutraceutical, indicating “Food, or part of a food, that provides medical or health benefits, including the prevention and/or treatment of a disease” [131]. This concept can be considered the evolution of the Hippocrates statement “Let food be the medicine and medicine be the food” [132]. On the basis of this definition, coumarin can be considered a nutraceutical with a solid scientific background. Unfortunately, nutraceuticals are still in the grey area between food, food supplements, and pharmaceuticals, and a shared and accepted definition of these products is still missing [133].

However, one could legitimately question whether the TDI established for conventional foods should also be applied when the same substance is used as a nutraceutical. Even more so considering that clinical trials clearly demonstrated that coumarin-induced hepatotoxicity is restricted to a small of subjects. In these cases, hepatotoxicity mostly occurred in a mild to moderate form. In most cases, patients showed transient elevation of transaminase levels which returned to normal during the treatment or immediately after. Furthermore, to our knowledge, no cases of liver failure have been reported and only one case of severe hepatotoxicity was assessed.

As previously discussed, several isoforms of the cytochrome p450 enzymes (CYPs) are involved in coumarin metabolism. In humans, coumarin is predominantly eliminated via 7-hydroxylation by p450 CYP2A6. Conversely, the coumarin-induced liver toxicity is related to the coumarin metabolite o-HPA deriving from CE via the alternative heterocyclic ring-splitting pathway. This seems to be the predominant pathway in patients that develop hepatotoxicity.

Several studies, confirmed by recent experiments performed on rat and human liver microsomes, showed that the enzymes CYP1A2 and CYP2E1 are involved in catalysing the ring-splitting route [134]. Interestingly, Miura et al. demonstrated that the inhibition of human CYP2E by furafylline results in a marked reduction of o-HPA plasma levels, compared to the control [135].

Interesting data come from computational studies that are thoroughly discussed in a recent review. These studies regarding the interaction of coumarin with CYPs enzymes show some of the possible mechanisms involved in the activation/inhibition of coumarin metabolic pathways that can help clarify its significance in toxicology [59].

Understanding the role of the enzymes involved in the coumarin metabolism and detoxification and identifying alterations in these enzymes can be useful to clarify the mechanisms underlying the metabolic activation of the CE pathway and to assess the risk in the population.

Analysis of CYP2A6 polymorphism, as previously reported, did not reveal a correlation with coumarin-induced hepatotoxicity. A study carried out to determine whether coumarin-associated liver dysfunction is genetically determined by polymorphism in CYP2A6 and impairment of the 7-hydroxylation of coumarin [90] demonstrated that here was no significant difference in the incidence of liver dysfunction between heterozygotes with CYP2A6*2, CYP2A6*3 and wild-type homozygotes. However, it is not to be excluded that polymorphism in other genes such as those coding for CYP2E1; CYP1A2 enzymes, as suggested by the results obtained by Miuru et al.; or those coding for ALDH, that is involved in the oxidative detoxification of o-HPA, could be involved in hepatotoxicity [34]. Indeed, an ALDH2 polymorphism is known to be related to a marked sensitivity to acetaldehyde, mainly in Asiatic populations [136]. Notably, a contingent combination of these is not to be excluded. Studies demonstrated that the expression of p450 genes is influenced by a combination of different factors including genetic polymorphisms, sex, age, ethnicity, health conditions, and induction by xenobiotics [137]. Thus, other factors such as the concomitant use of other drugs that could interfere with coumarin metabolism, previous liver injuries, smoking habits [134], and alcohol consumption could contribute to increased coumarin toxicological risk and must be considered. Indeed, it has been demonstrated that human CYP2A6 expression is induced by alcohol [138].

As coumarin-induced hepatotoxicity is a multifactorial outcome, the identification of risk factors should follow a multidisciplinary approach. For this purpose, current advances in biotechnology and computational models could be exploited to improve pharmacogenetics and genetic screening data obtained in the past studies. Furthermore, these could be joined into a new approach methodologies system (NAMs). An interesting and innovative approach, currently limited to cosmetic ingredients toxicity assessment, is the Next Generation Risk Assessment (NGRA). Interestingly, a recent Next-Generation Risk Assessment Case study has been reported specifically for coumarin in cosmetic products [139].

In association with data obtained from conventional experimental methods, extending NGRA to the risk assessment of products intended for therapeutic use could be a possible future strategy to improve our knowledge of coumarin toxicity.

## 6. Conclusions

Coumarin, used for different purposes, in monotherapy as well as in combination with other products, is safe and well-tolerated in most patients, with only a small subgroup of subjects showing signs of mild to moderate hepatotoxicity. However, the rare occurrence of these outcomes led to limitations and/or bans on the use of coumarin as a therapeutic in several countries. Further studies are needed to identify individuals at risk of developing hepatotoxicity. In this context, two areas of particular interest are the analysis of polymorphisms of genes coding for enzymes involved in the metabolism and/or detoxification of coumarin, including ALDH and cytochrome P450 isoforms (also different from CYP2A6), and the identification of the environmental factors involved. In addition, a useful parameter could be the evaluation of the type and concentration of coumarin metabolites in urine during the first weeks of treatment, that could indicate possible variations in metabolic pathways. Importantly, due to the multifactorial nature of coumarin-induced hepatotoxicity, an integrated approach (e.g., NAMs) should be applied to improve the current knowledge on this topic.

In conclusion, the aim of this work is to stimulate research and advancements on coumarin hepatotoxicity knowledge since the recent literature in this field is very limited and should be implemented especially considering current biotechnological advancements. Early identification of patients at risk of hepatotoxicity could allow the possibility of safely exploiting the health-promoting effects of coumarin.

## Figures and Tables

**Table 1:** Coumarin safety and tolerability in clinical trials.

Number of Patients	Disease	Coumarin Dose	Cotreatment with Other Drugs	Hepatotoxic Effects (Number of Patients)	References
7	Melanoma	100 mg/day	No	No	Zanker et al. (1984) [101]
17	Cancer	100 mg/day	Cimetidine 1 g/day	No	Nolte et al. (1987) [116]
13	Melanoma	50 mg/day	No	No	Thornes et al. (1989) [102]
42	Lymphoedema secondary to filiriasis	400 mg/day	Carbamazine 6 mg/Kg per day	No	Jamal et al. (1989) [100]
39	Lymphoedema secondary to filariasis	No	Carbamazine 6 mg/Kg per day	No	
47	Lymphoedema secondary to filariasis	400 mg/day	No	No	
50	Cancer	100 mg/day	Cimetidine 1.2 g/day	No	Dexeus et al. (1990) [104]
38	Renal cell carcinoma	100 mg/day	Cimetidine 400 mg/day	No	Kokron et al. (1991) [117]
48	Prostate cancer	3 g/day	No	Asymptomatic transaminase elevation (3)	Mohler et al. (1992) [112]
31	Lymphoedema secondary to breast cancer	400 mg/day	No	No	Casley-Smith et al. (1993a) [113]
45	Chronic lymphatic filariosis	400 mg/day	No	No	Casley-Smith et al. (1993) [114]
91	Cancer	100 mg/day	Cimetidine 300 mg/day	No	Marshall et al. (1994) [37]
1106	Lymphoedema	400 mg/day	No	Mild hepatotoxicity regressed after ceased treatment (1)	Casley-Smith et al. (1995) [79]
1 (Case report)	Lymphoedema	400 mg/day	No	Severe hepatotoxicity (1)	Morrison and Welsby (1995) [80]
30	Chronic lymphoedema	400 mg/day	No	No	Chang et al. (1996) [123]
2	Lymphoedema	90 mg/day	No	Hepatitis with favourable outcome (2)	Koch et al. (1997) [82]
2173	Cancer/chronic infections	100–300 mg/day	No	Serum transaminase elevation at 1 to 15 g of total dose (8)	Cox et al. (1989) [103]
38	Lymphoedema secondary to breast cancer	90 mg/day	No	SGPT elevation (1)	Burgos et al. (1999) [115]
39	Lymphoedema secondary to breast cancer	135 mg/day	No	SGPT elevation (1)	Burgos et al. (1999) [115]
48	Cancer	90 mg/day	Troxerutin 540 mg/day	No	Grötz et al. (2001) [118]
114	Chronic venous insufficiency	90 mg/day	No	Mild changes in liver function parameters (4.9%) compared to controls (2.1%)	Vanscheidt et al. (2002) [119]; Schmeck-Lindenau et al. (2003) [120]
60	Lymphatic oedema of lower limbs	10 mg/day	No	No statistical differences compared to controls	Lessiani et al. (2015) [121]

## Data Availability

Not applicable.

## Sample Availability

Samples of the compounds are not available from the authors.

## Abbreviations

ALT	alanine aminotransferase
ULN	upper limits of normal
CYPs	cytochrome P450 family
7-HC	7-hydroxycoumarin
7-HCG	7-hydroxycoumarin-glucuronide
CE	3,4-epoxycoumarin
o-HPA	o-hydroxyphenylacetaldehyde
o-HPAA	o-hydroxyphenylacetic acid
GSH	glutathione
SGPT	serum glutamic pyruvic transaminase
AST	aspartate transaminase
gamma-GT/GGT	gamma-glutamyl transpeptidase
TDI	Tolerable Daily Intake
EFSA	European Food Safety Authority
NOAEL	No Observed Adverse Effect Level
BfR	German Federal Institute for Risk Assessment
ALDH	aldehyde dehydrogenase
NAMs	new approach methodologies system
NGRA	Next Generation Risk Assessment
